# Expansion of tumor-infiltrating lymphocytes (TIL) from human pancreatic tumors

**DOI:** 10.1186/s40425-016-0164-7

**Published:** 2016-10-18

**Authors:** MacLean Hall, Hao Liu, Mokenge Malafa, Barbara Centeno, Pamela J. Hodul, José Pimiento, Shari Pilon-Thomas, Amod A. Sarnaik

**Affiliations:** 1 Department of Immunology, H Lee Moffitt Cancer Center and Research Institute, Tampa, FL USA; 2 Gastrointestinal Oncology Program, H Lee Moffitt Cancer Center and Research Institute, Tampa, FL USA; 3 Cutaneous Oncology Program, H Lee Moffitt Cancer Center and Research Institute, 10920 N. McKinley Dr, Tampa, FL 33612 USA

**Keywords:** Pancreatic cancer, T cells, Adoptive cell therapy, Tumor infiltrating lymphocytes (TIL)

## Abstract

**Background:**

We evaluated whether tumor infiltrating lymphocytes (TIL) could be expanded from surgically resected tumors from pancreatic cancer patients.

**Methods:**

Tumors were resected from pancreatic cancer patients. Tumors were minced into fragments and cultured in media containing high dose interleukin-2 (IL-2) for up to 6 weeks. T cell phenotype, activation markers, and reactivity were measured.

**Results:**

TIL expansion was measured in 19 patient samples. The majority of these TIL were CD4^+^ T cells and were highly activated. Purified CD8^+^ T cells produced IFN-γ in response to HLA-matched pancreatic tumor targets. PD-1 blockade and 4-1BB stimulation were demonstrated as effective strategies to improve effective TIL yield, including the production of tumor-reactive pancreatic TIL.

**Conclusions:**

TIL expanded from pancreatic tumors are functional and able to respond to pancreatic tumor associated antigens. PD-1 blockade, 41BB stimulation, and CD8^+^ T cell enrichment are effective strategies to improve TIL yield and tumor reactivity. These results support the development of adoptive cell therapy strategies using TIL for the treatment of pancreatic cancer.

**Electronic supplementary material:**

The online version of this article (doi:10.1186/s40425-016-0164-7) contains supplementary material, which is available to authorized users.

## Background

Pancreatic adenocarcinoma is the fourth-leading cause of cancer-related mortality in the United States. Patients diagnosed with this disease face a 5-year survival rate of less than 5 %, and the only available treatments, surgery, chemotherapy and chemoradiation, have shown limited effectiveness [1, 2]. Only a small fraction (20 %) of these patients are even eligible for surgery with curative intent, and most will develop recurrent disease within 2 years of definitive therapy [3]. Therefore, alternative treatment strategies are urgently needed.

Recent successes in immunotherapy for the treatment of metastatic melanoma have resulted in its application to other types of cancer. Specifically, adoptive cell therapy (ACT) is a particularly promising approach that utilizes endogenous tumor-infiltrating lymphocytes (TIL), which are expanded in vitro from a surgically resected tumor and then re-infused back into the patient. This therapy for metastatic melanoma patients is associated with a 20 % complete response lasting beyond 3 years [4]. In patients with gastrointestinal (GI) tumors, infiltration of CD3^+^ T cells is associated with a higher rate of progression free survival [5], and pancreatic adenocarcinomas containing both CD4^+^ and CD8^+^ T cells correlated with an improved prognosis and significantly greater 5-year survival for these patients [6–8]. Therefore, there is evidence of a host T cell immune response in patients with pancreatic adenocarcinoma, supporting the potential application of ACT using TIL for this cancer histology.

Correspondingly, there is no shortage of studies demonstrating that the tumor microenvironment of pancreatic adenocarcinoma is inherently immunosuppressive, with a vast array of mechanisms to escape immune surveillance. These include co-inhibitory ligands, such as PDL1 and PDL2, which directly interact with T cells to dampen their effector response [9–12], regulatory T cells [13–17], reduced antigen presentation [18], and suppressive cytokines [19–21]. Additionally, activated TIL upregulate checkpoint molecules such as PD-1, which may serve to dull the intensity of the inflammatory response and induce tolerance toward tumor antigens [22].

Despite all of these factors, TIL expanded in vitro to large numbers have the potential to be reprogrammed as effectors of a productive anti-tumor response [4]. In fact, it has recently been demonstrated that PD-1^+^ TIL represent the repertoire of clonally expanded tumor-reactive cells in melanoma [23]. Identifying and targeting enriched, tumor-specific TIL subsets in vitro and in vivo is an active area of research intended to augment TIL growth and function in order to improve the efficacy of ACT.

In the present study, we successfully expanded TIL from 19 patients with pancreatic adenocarcinoma in the presence of high dose IL-2. The majority of these TIL were CD4^+^ T cells and were highly activated. Both PD-1 blockade and 4-1BB agonism led to improved TIL yields. Enrichment of CD8^+^ T cells was an effective strategy to measure tumor-reactive pancreatic TIL. These results support the use of TIL expanded from pancreatic tumors in ACT strategies for patients with pancreatic adenocarcinoma.

## Results

### Pancreatic TIL are predominantly CD4

**Fig. 1:**
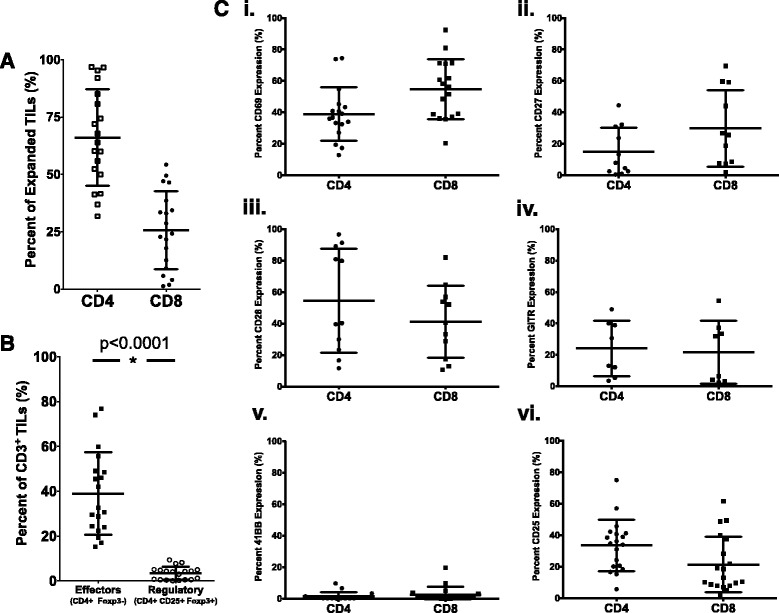
Characterization of pancreatic TIL after expansion. **a** Expanded pancreatic TIL were characterized phenotypically by flow cytometry for CD4 and CD8 expression on CD3+ lymphocytes (mean % ± SD). *n* = 19 (**b**) TIL were surface stained with CD3, CD4, CD8, and CD25, then further characterized by intracellular staining with Foxp3. Data are reported as mean % ± SD. *p* < 0.0001 as determined by Wilcoxon matched pairs signed rank test. *n* = 19. **c** TIL were additionally surface stained for markers of activation and antigen experience CD69 (*i*, *n* = 17), CD27 (*ii*, *n* = 11), CD28 (*iii*, *n* = 11), GITR (*iv*, *n* = 8), 4-1BB (*v*, *n* = 18), and CD25 (*vi*, *n* = 19) on CD4+ and CD8+ cells within the CD3+ lymphocyte population. Summary data are reported as mean % ± SD

In order to establish the feasibility of isolating and expanding TIL from pancreatic adenocarcinomas, we adapted our previous experience with melanoma to establish pancreatic TIL cultures [24]. Tumors were surgically resected from 20 different patients with pancreatic cancer at the Moffitt Cancer Center and used to set up tumor fragment cultures for the isolation and propagation of TIL. Of note, the volume of tumor received in the laboratory after pathological analysis was considerably smaller compared to those typically obtained to derive melanoma TIL. This resulted in an average of 14.3 tumor fragments from resected pancreatic cancer specimens from which TIL were initially propagated, compared to over 48 fragments from a typical melanoma resection. Despite this, TIL were successfully isolated from at least one plated fragment in 19 of the 20 patient tumors (95 %) in the presence of high dose IL-2 (6000 IU/mL) after 3 to 6 weeks of culture (Table 1 and Additional file 1: Table S1) and were predominantly CD4^+^ T cells. TIL yield varied between patient samples with an average yield of 1.79x10^7^ TIL from an average of just over 14 fragments per patient. Four samples of the 17 (23.5 %) measured gave rise to at least 25 million TIL, the minimum number required for initiation of a clinical scale rapid expansion protocol (REP). Extrapolated to the 48 fragments typically set up during a clinical scale expansion, the average yield increased to over 83 million TIL, with nine of 17 (52.9 %) samples meeting the REP initiation threshold. The majority of these expanded TIL were CD4^+^ (66.1 ± 21.0 %), while CD8^+^ T cells comprised a mean of 25.6 ± 17.0 % (Fig. 1a). The remaining analyzed CD3^+^ TIL not within these single positive gates were predominantly double negative cells. Importantly, pancreatic TIL were also capable of REP, the second phase of pre-infusion TIL growth. Three patient samples were subjected to the full, two week REP which resulted in an average fold expansion of 964 (Additional file 1: Table S2), which compared similarly to our experience in melanoma TIL where ~1000 fold expansion is observed during the REP. Twelve additional samples underwent an 8 day mini-REP and exhibited a 93-fold expansion on average over that time (*n* = 12, data not shown). This data demonstrate that generating sufficient TIL for adoptive cell therapy from pancreatic adenocarcinoma fragments is possible given the procurement of adequately sized specimens needed to yield the high numbers of TIL required to initiate the REP.

**Table 1:** TILs from Pancreatic Adenocarcinoma are Predominantly CD4+

Patient	CD4+	CD8+	Fragments plated	TIL yield	Yield per 48 fragments
1	31.8	54.3	4	4.97E + 05	5.96E + 06
2	95.4	4.04	9	1.40E + 08	7.47E + 08
3	96.9	1.90	24	1.13E + 07	2.26E + 07
4	96.6	1.34	24	6.40E + 05	1.28E + 06
5	64.0	33.5	23	3.78E + 07	7.89E + 07
6	72.0	21.7	24	2.52E + 07	5.04E + 07
7	60.2	33.0	33	5.32E + 06	7.74E + 06
8	74.5	22.8	12	1.42E + 06	5.68E + 06
9	92.1	5.79	36	3.64E + 07	4.85E + 07
10	41.3	46.5	10	5.90E + 06	2.83E + 07
11	^a^	^a^	9	N/A	N/A
12	52.4	34.4	24	ND	ND
13	85.1	12.6	1	2.92E + 06	1.40E + 08
14	49.9	17.9	12	1.80E + 06	7.20E + 06
15	36.9	49.5	5	1.34E + 06	1.29E + 07
16	55.8	38.6	2	6.90E + 05	1.66E + 07
17	60.1	24.2	1	ND	ND
18	67.9	28.7	7	2.37E + 07	1.63E + 08
19	80.7	9.02	14	1.89E + 06	4.68E + 07
20	41.6	47.1	12	7.50E + 06	7.50E + 06
Mean	66.1	25.6	14.3	1.79E + 07	8.31E + 07
SD	21.0	17.0	10.5		

All TIL analyzed are pre-Rapid Expansion Protocol (REP)

^a^ TIL growth not observed; *N/A* not applicable, *ND* no data

### Pancreatic TIL have an activated phenotype

To characterize the phenotype of these expanded pancreatic TIL, we examined the expression of markers involved in the activation, differentiation and function of T cells. Since the majority of TIL were CD4^+^, we investigated the presence of regulatory T cells (T_regs_) by intracellular staining for FOXP3 on the CD4^+^CD25^+^ population. We found that the frequency of CD4^+^CD25^+^FOXP3^+^ T_regs_ is quite low (~4 %) in expanded pancreatic TIL as a proportion of the total CD3^+^ population (Fig. 1b). TIL were nearly entirely CD45RO^+^ CCR7^−^, characteristic of antigen-experienced effector T cells (data not shown). Approximately 50 % of CD8^+^ cells and 40 % of CD4^+^ cells expressed CD69 (Fig. 1c *i*), indicating an efficient and sustained T cell stimulation [25]. TIL also exhibited a low level of CD27 expression (Fig. 1c *ii*), a marker known to be down-regulated as T cells approach terminal differentiation [26]. We further characterized the phenotype of these cultured TIL and found that more than 40 % of CD8^+^ cells expressed the co-stimulatory marker CD28 (Fig. 1c *iii*) and more than 20 % cells expressed GITR (Fig. 1c *iv*), a receptor crucial for the proliferation of activated CD8^+^ T cells [27]. Additionally, less than 5 % of TIL were 4-1BB^+^, a co-stimulatory marker that is expressed on activated T cells after TCR engagement (Fig. 1c *v*) [28]. IL-2 receptor alpha (CD25), which is up-regulated after TCR stimulation, was expressed in greater than 30 % of CD4^+^ TIL and 20 % of CD8^+^ TIL (Fig. 1c *vi*) [29]. Data for each of these phenotypic markers can be found in Additional file 1: Table S1 on a per patient basis. Notably, as evaluated on a small cohort of samples, these phenotypic indicators of activation were largely unchanged, showing no significant difference through the two-week REP (Additional file 1: Figure S2). Overall, pancreatic adenocarcinoma TIL that were successfully expanded from tumor fragments were found to express numerous markers of activation and antigen experience and were comprised of a low frequency of Tregs.

### PD-1 blockade increases expansion of pancreatic TIL

**Fig. 2:**
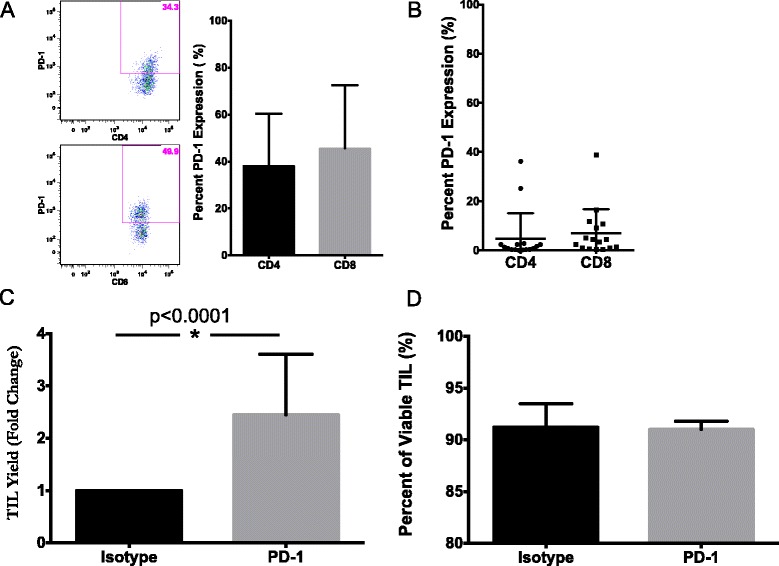
PD-1 inhibition yields increased pancreatic TIL. **a** Expression of PD-1 on TIL in digested pancreatic tumor samples was measured by flow cytometry. Representative data shown in flow plots as well as summary data of mean ± SD (*n* = 4). **b** After expansion, TIL were surface stained for CD3, CD4, CD8, and PD-1 and analyzed for PD-1 expression on CD3+ CD4+ or CD3+ CD8+ lymphocytes. Data are reported as mean % ± SD (*n* = 16). Pancreatic TIL were then propagated from fragments of pancreatic adenocarcinoma specimens in 6000 IU/mL rhIL-2 in the presence of 10 μg/mL α-PD-1 or the isotype control (IgG) antibody. After 2 weeks, total TIL were counted (**c**), and assessed for viability (**d**). Fold change was normalized to the isotype control. Column graph displays mean values with positive SD shown. Percentages present the corresponding frequency in the PD-1 or control group (*n* = 3 patients). Statistical significance was determined by the Wilcoxon signed rank test

In addition to the above phenotypic markers, we also stained for the presence of PD-1 on pancreatic TIL. PD-1 is a co-inhibitory receptor that restricts T cell activity and has recently been shown to be specifically present on tumor-reactive TIL [23, 30]. TIL from digested tumor samples analyzed by flow cytometry displayed relatively high levels of PD-1, as over 30 % of CD4+ TIL (38.2 ± 22.1 %) and 40 % of CD8^+^ TIL (45.7 ± 26.8 %) were positive for surface PD-1 (Fig. 2a). In contrast, the level of PD-1 expression was low on expanded pancreatic TIL, with a frequency of 4.8 % for CD4^+^ T cells and 6.9 % for CD8^+^ T cells (Fig. 2b).

The detection of PD-1 expression on freshly isolated TIL prompted an investigation of whether the blockade of PD-1 inhibitory signaling in early culture could affect the outcome of pancreatic TIL growth. An antagonistic PD-1 antibody was added at the initiation of TIL culture to determine its effect on TIL propagation from pancreatic tumors. This was compared to culture of TIL in the presence of an isotype control antibody. Addition of the isotype antibody had no effect on TIL expansion (data not shown). The absolute number of TIL from three patients dramatically increased after PD-1 blockade (2.5 fold), while frequency of cell death in comparison to the isotype control was unchanged (Fig. 2c, d). Pancreatic TIL cultured in the presence of anti-PD-1 antibody from a single patient produced significantly more IFN-γ in the presence of a HLA-matched pancreatic tumor line as compared to TIL cultured with an isotype control (Additional file 1: Figure S1). These data suggested that the inhibition of PD-1 signaling can augment the propagation of pancreatic TIL from tumor fragments and increase the expansion of pancreatic tumor-specific T cells.

### Targeting 4-1BB increases expansion of pancreatic TIL

**Fig. 3:**
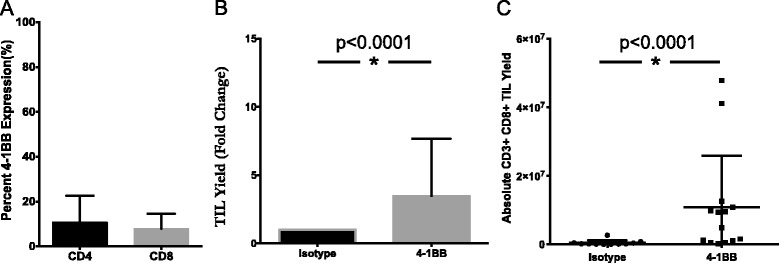
Addition of 4-1BB agonist improves yield of CD8^+^ pancreatic TIL. **a** Pancreatic tumor digests were assessed for 4-1BB expression by flow cytometry. The data represent mean + SD (*n* = 3). Separately, fragments of pancreatic tumors were initially plated in TIL CM containing 6000 I.U./mL IL-2 and 10 ug/mL anti-4-1BB antibody or IgG4 isotype control. TIL were subsequently cultured according to standard techniques, with the addition of 1 ug/mL anti-4-1BB or isotype. **b** Viable TIL were counted by trypan blue exclusion after pre-REP expansion and reported as a fold change over isotype control for each patient total (*n* = 3 patients). Significance was determined by the Wilcoxon signed rank test. **c** Pancreatic TIL were then analyzed by flow cytometry for CD3, CD4, CD8 expression on live lymphocytes. Absolute numbers were derived from cell counts and percentages based on FACS analysis on a per fragment basis. Statistical significance was determined by the Mann-Whitney Test (*n* = 3 patients). Data are reported as mean % ± SD

Similarly, the effect of an agonistic 4-1BB antibody on TIL growth was determined through its addition during TIL expansion. In digested pancreatic tumor samples, 10.8 % of CD4^+^ TIL and 7.9 % of CD8^+^ TIL expressed 4-1BB (Fig. 3a). Tumor fragments were cultured in IL-2 in the presence or absence of anti-4-1BB antibody. On a per fragment basis, both the absolute TIL yield and the number of CD3^+^CD8^+^ lymphocytes were significantly augmented in the 4-1BB treated group when compared to the isotype control. Overall, a 3.5-fold expansion was measured (Fig. 3b), while a 23-fold difference in the CD8^+^ TIL resulted from the addition of agonistic 4-1BB (Fig. 3c). These results support the targeting of 4-1BB as a strategy to selectively expand CD8^+^ T cells from pancreatic tumors, which have been shown to be essential to the anti-tumor response [31].

### Functional potential of pancreatic TIL

**Fig. 4:**
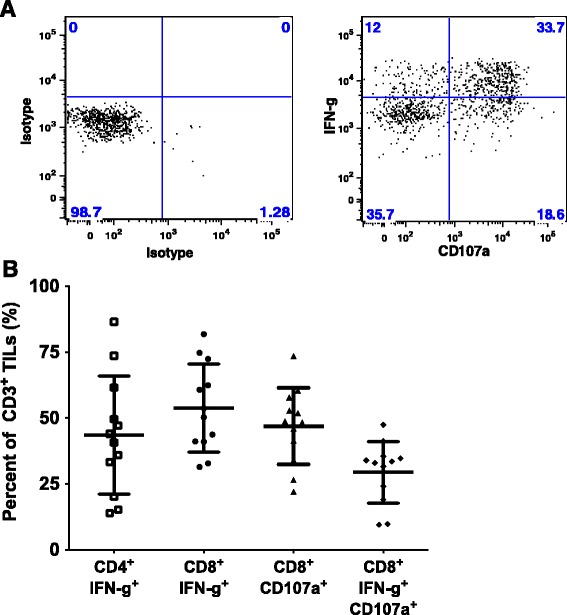
Stimulated pancreatic TIL display effector function. **a** Propagated TIL were stimulated with 25 ng/mL of PMA and 500 nM ionomycin for 18 h, then stained for CD3, CD4, CD8, and CD107a or the corresponding isotype antibodies followed by intracellular staining for IFN-γ and analyzed by flow cytometry. All cells stained with the isotype control were stimulated under the same conditions. Representative FACS plots gated on CD3^+^ CD8^+^ TIL are shown. **b** Scatter plots display the percentage of each indicated population for individual patient data. Each analysis refers to the percentage of IFN-gamma (IFN-g) + and/or CD107a + cells within the CD4 or CD8 gates of the CD3^+^ lymphocytes. Error bars represent mean % ± SD (*n* = 12)

To investigate whether these expanded pancreatic TIL are capable of responding to extracellular stimulation, TIL were stimulated with PMA and ionomycin. After 18 h, we observed a clear increase in surface expression of CD107a, which is associated with degranulation and cytotoxic activity of T cells, and accumulated intracellular IFN-γ, an important effector of the TIL anti-tumor response [32, 33]. We found that more than 50 % of CD8^+^ pancreatic TIL were positive for IFN-γ expression, and greater than 70 % of CD8^+^ TIL were capable of producing either CD107a or IFN-γ (Fig. 4a, b). Under these same conditions, greater than 80 % of melanoma TIL were able to present IFN-γ (data not shown). These data suggest that expanded pancreatic TIL contain the functional potential to elicit an anti-tumor response.

**Fig. 5:**
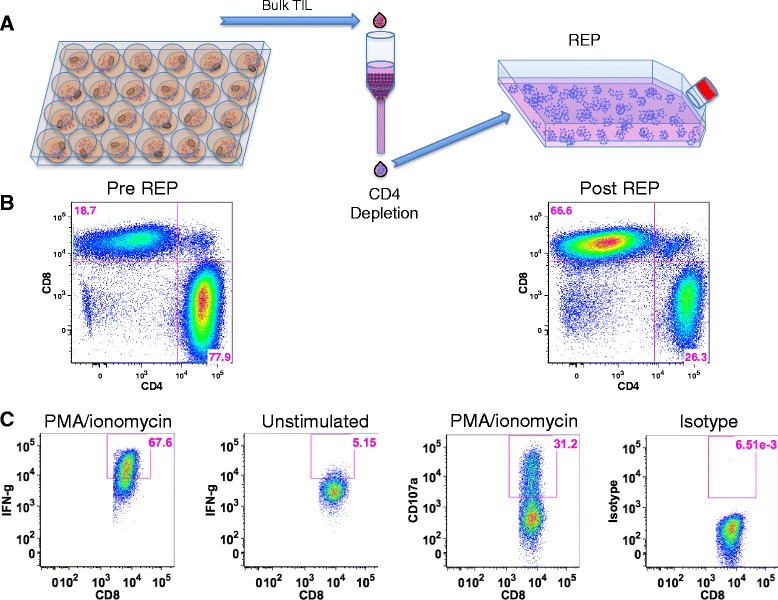
CD8^+^ pancreatic TIL maintain effector function after selective expansion. **a** CD8^+^ T cells were enriched through magnetic depletion of CD4^+^ cells from bulk TIL and subject to the rapid expansion protocol (REP). **b** Representative FACS plots of CD3^+^ TIL were analyzed for CD4 and CD8 expression before and after the procedure described in **a. c** CD8-enriched TIL were then stimulated with 25 ng/mL of PMA and 500 nM ionomycin and analyzed for surface CD107a and intracellular IFN-gamma (IFN-g) by flow cytometry

As reported above, the majority (66 %) of expanded pancreatic TIL were found to be CD4^+^ (Fig. 1a). Although these T cells were capable of producing IFN-γ at a similar level to CD8^+^ TIL in response to stimulation (Fig. 4b), the tumor-specific immune response is thought to be mediated primarily by CD8^+^ cells [34]. To help define the function of CD8^+^ pancreatic TIL, we enriched CD8^+^ cells from whole TIL by depletion of CD4^+^ cells. The remaining CD8^+^ TIL were then expanded following the mini-REP, as diagrammed in Fig. 5a. Selective expansion of CD8^+^ T cells in the mini-REP prevented their dilution by CD4^+^ T cells, and allowed an increased opportunity to capture CD8^+^ T cell tumor reactivity through a co-culture assay. The CD8^+^ TIL were enriched from 19 % to 67 % and remained predominantly CD8^+^ throughout this substantial expansion (Fig. 5b). Furthermore, these post-REP CD8^+^ TIL retained their effector capacity in response to stimulation with PMA and ionomycin (Fig. 5c), as the majority of TIL expressed intracellular IFN-γ and nearly one third were CD107a^+^.

### CD8+ pancreatic TIL are tumor specific

**Fig. 6:**
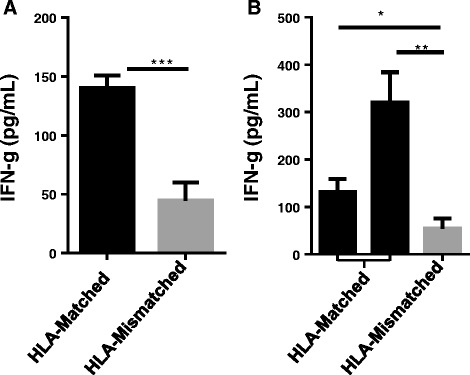
Pancreatic TIL produced tumor specific immune response. CD8-enriched post-REP TIL from two patients were cultured with HLA-matched (in *black*) or mismatched (in *gray*) pancreatic cancer cell lines at a 1:1 ratio. **a** The patient was HLA-A typed as A01/24 and matched with the MiaPaca-2 (A24) cell line, and mismatched with the Panc-1 (A02/11) pancreatic tumor cell lines. **b** The patient was HLA-A typed as A02/24 and matched with the CFPAC-1 (A02/03) and Panc-1 (A02/11) pancreatic tumor cell lines, and mismatched with HPAFII (A01/11) tumor cell line. IFN-gamma levels in supernatants were measured after 48 h and reported as mean ± SEM and statistical significance was determined by the student’s *t*-test. *, *p* < 0.05, **, *p* < 0.01, ***, *p* < 0.001

To further evaluate whether pancreatic TIL have tumor specificity, CD8^+^ post-REP TIL were cultured with HLA-matched pancreatic cell lines. The amount of IFN-γ released in co-culture was measured after 48 h. Pancreatic TIL from two individual patients produced significantly more IFN-γ in the presence of HLA-matched pancreatic tumor lines as compared to HLA-mismatched tumor (Fig. 6a, b). This data demonstrates that tumor-reactive CD8^+^ T cells are present within the repertoire of pancreatic TIL and are functional against shared pancreatic tumor antigens in an HLA-dependent manner.

## Discussion

The development of novel therapeutic approaches for pancreatic adenocarcinoma is paramount to the successful treatment and recovery from this aggressive cancer. In the age of personalized cancer medicine, adoptive cell therapy (ACT) using tumor-infiltrating lymphocytes (TIL) represents a unique opportunity to exploit both the diversity and specificity of a patient’s immune system. The CD8^+^ T cells within this population represent an endogenous polyclonal repertoire of TCRs specific and comprehensive for the array of tumor-associated antigens (TAAs) and unique neoantigens present [35]. Within the appropriate setting, these infiltrates are capable effectors of a targeted anti-tumor response that can be both durable and complete [4].

In the present study, we explored the feasibility of expanding TIL from resected pancreatic adenocarcinomas. ACT is dependent upon both the quantity and quality of the lymphocytes recovered from the original source. Importantly, we showed that TIL can be expanded from surgically resected pancreatic tumors and REP’d to clinically relevant metrics. Given the inherent constraint of only one in every five patients with pancreatic cancer as a candidate for surgery for curative intent, it is significant that nearly all of the tumors in this study contained lymphocytes capable of in vitro propagation. In addition, a recent study has also demonstrated the feasibility of expanding functional TIL from pancreatic tumor samples [36].

Excess (not required for pathologic analysis), surgically resected pancreatic adenocarcinomas received in the laboratory for TIL generation were characteristically small in size, restricting the supply of starting material crucial to culturing TIL from tumor fragments. It has previously been reported that the density of infiltrating lymphocytes is also inferior in GI tumors when compared to melanoma, leading to an increased time required for expansion [28]. These drawbacks are important to consider given that one central concern of ACT is the necessary delay between surgery and infusion. Pretreatment of patients, such as with immune checkpoint inhibitor therapies, prior to surgical resection in order to enrich tumors for specific lymphocyte populations should be investigated as a means to overcome this limitation.

T cells localized to the pancreatic tumor microenvironment have long been characterized as both intrinsically and extrinsically suppressed, frequently attributed to the presence of T_regs_ [14, 37, 38]. Importantly, in the current study after expansion in IL-2, pancreatic TIL were predominantly activated T cells, before and after REP, and presented markers of antigen experience, including CD69 and CD45RO. Furthermore, we demonstrated that high doses of IL-2 in vitro did not polarize the pancreatic TIL cultures toward a T_reg_ phenotype, but may have influenced the observed activated phenotype. A relatively high T_eff_ to T_reg_ ratio was established, which is thought to be critical for effective immunosurveillance of tumors [37, 38]. This result highlights the importance of releasing pancreatic TIL from their naturally inhibitory tumor microenvironment and supports the notion that pancreatic TIL have a plastic phenotype that can be polarized toward an effector function. Therefore, our data on pancreatic TIL support previous reports that TIL from GI tumors resemble the activated state of TIL derived from melanomas [28].

We found that the inhibitory molecule PD-1 was expressed at substantial levels on minimally cultured pancreatic TIL, within the range of PD-1 expression reported on TIL from other GI tumors [28]. The presence of PD-1, which is thought to be preferentially expressed on a comprehensive repertoire of tumor-reactive TIL, confirmed that these T cells were antigen-experienced and demonstrated the need to isolate these TIL from potential sources of ligand inhibition [23, 30]. Moreover, we were able to capitalize on the surface expression of PD-1 through the addition of a blocking antibody to the TIL culture and observed a substantial increase in the expansion of TIL. This data suggest that disrupting PD-1 signaling during ex vivo TIL culture may selectively expand tumor-reactive T cells, but this requires further investigation before any conclusions can be drawn. Additionally, PD-1 expression on TIL is known to be transient during culture with IL-2, as we also observed (Fig. 2b), potentially highlighting the limited window to employ this strategy for augmenting TIL growth [30].

We also investigated the effects of an agonistic 4-1BB antibody on its ability to improve the yield of pancreatic TIL as we have previously observed this in TIL derived from melanoma [39]. TIL expanded in media supplemented with anti-4-1BB demonstrated a significant increase in the absolute number of TIL produced on a per fragment basis. Furthermore, these TIL were predominantly CD8^+^ T cells with a 32-fold difference in yield of this population compared to control cultures. As 4-1BB positive TIL are thought to correspond to those T cells undergoing recent TCR engagement, it is possible that this selective expansion of CD8^+^ lymphocytes represents the population of tumor resident TIL specific for expressed tumor antigens on the surface of pancreatic adenocarcinoma [28].

It is widely accepted that CD8^+^ T cells are instrumental for an anti-tumor response, but the involvement of CD4^+^ T cells is far less clear and potentially disruptive [31, 40, 41]. In one murine study, the presence of TNFα and IL-17-producing CD4^+^ T cells in pancreatic cancer was associated with relatively aggressive disease [42]. For this reason, we chose to isolate the CD8^+^ pancreatic TIL to use as effectors in tumor reactivity assays. A major difficulty for the translation of TIL therapy into pancreatic patients is the lack of autologous tumor for the evaluation of tumor-specific reactivity. This required a reliance on HLA-matched pancreatic tumor lines, known for their relative lack of shared antigens, as TIL targets [35]. Importantly, we were able to demonstrate a significant and specific, HLA restricted immune response to matched tumor targets using this strategy. Previous studies have shown that an immune response toward shared pancreatic cancer antigens can be elicited, and our results confirm that TIL expanded from pancreatic tumors are functional when re-stimulated with HLA-matched tumor targets [7, 13, 43–48]. A recent study demonstrated that TIL expanded from pancreatic tumors recognized shared pancreatic tumor antigens, including NY-ESO-1, survivin, and mesothelin [36]. While reactivity to shared antigens can be measured in a subset of pancreatic TIL samples, it has been shown that patient with metastatic GI malignancies have unique mutations, and anti-tumor T cell responses are targeted to neoantigens specific to each individual patient [35]. Moving forward, it will be important to develop effective methods to establish autologous tumor targets. This would also allow for more complete evaluation of the TIL compartment, including CD4^+^ TIL, which have been previously demonstrated to recognize autologous tumor [41].

## Conclusions

TIL were readily isolated and expanded from pancreatic tumors and characterized by a predominantly activated phenotype, rather than the suppressor phenotype previously reported to be present in the tumor microenvironment. The ability of pancreatic TIL to react to tumor antigen stimulation indicates that this population has the capacity to effect a tumor-specific response. Therefore, ACT with TIL is a therapeutic strategy that should be further investigated for pancreatic adenocarcinoma, a cancer with high lethality and limited treatment options.

## Methods

### TIL culture

Pancreatic tumors were minced into ~1 mm^3^ fragments, placed in 24 well plates with 2 mL of TIL media containing IL-2, and pancreatic TIL were allowed to extravasate from the tissue. If available, excess tissue was physically and enzymatically digested as described below. Alternatively, 48 well plates were used following the same procedure, using 1 mL of TIL media and IL-2. TIL were expanded in vitro for 3–6 weeks in 6000 I.U./mL IL-2 (Proleukin, Novartis, Emeryville, CA) per mL of complete TIL media (TIL-CM) consisting of RPMI 1640, 2.05 mM L–glutamine (HyClone, Thermo Fisher Scientific, Waltham, MA), 10 % heat-inactivated human AB serum (Omega Scientific, Tarzana, CA), 55 μM 2-mercaptoethanol (Invitrogen), 50 μg/mL gentamicin (Invitogen), 100 I.U./mL penicillin, 100 μg/mL streptomycin, and 10 mM HEPES Buffer (Mediatech, Manassas, VA) in 24 or 48 well plates. Half of the media was replaced every 2 to 3 days or wells were split when 80 % confluent. For TIL generation in the presence of costimulatory antibodies, TIL were propagated as above with the addition of an antagonistic human PD-1 antibody (Nivolumab, 10 μg/mL) or with an agonistic human 4-1BB antibody (Urelumab, 10 μg/mL at initiation, followed by continued supplement with 1 μg/mL at each feeding or splitting as above) generously provided by Drs. Alan Korman and Maria Jure-Kunkel (Bristol Myers Squibb, Princeton, NJ).

### Rapid expansion protocol (REP)

In a GREX10 flask (Wilson Wolf), 5.0 × 10^5^ pancreatic TIL were stimulated with 30 ng/mL human anti-CD3 (OKT3, Ortho Pharmaceutical, Raritan, NJ) in the presence of 1.0 × 10^8^ irradiated (5000 rad) allogenic PBMC feeder cells. TIL were cultured in 20 mL of 50 % REP Media I (described below) and 50 % AIM V (Invitrogen) supplemented with 3000 I.U./mL rhIL-2. On day 4, 10 mL of the same media was added to the GREX flask. On day 7, cultures were counted and split as necessary and the remaining volume in the flask was filled with AIM V media supplemented with 3000 I.U./mL rhIL-2. After 14 days, TIL were collected and counted and rested in pre-REP culture conditions prior to future analysis.

### Mini rapid expansion protocol (mini-REP)

In a T25 flask, 1.43 × 10^5^ pancreatic TIL were stimulated with 30 ng/mL human anti-CD3 (OKT3, Ortho Pharmaceutical, Raritan, NJ) in the presence of 2.9 × 10^7^ irradiated (5000 rad) allogenic PBMC feeder cells. TIL were cultured in REP Media I comprised of RPMI 1640, 2.05 mM L–glutamine (HyClone, Thermo Fisher Scientific), 10 % heat-inactivated human AB serum (Omega Scientific), 55 μM 2-mercaptoethanol (Invitrogen), and 10 mM HEPES Buffer (Mediatech). On day 5, 70 % of the media was replaced with REP Media II comprised of a 1:1 (v:v) mixture of REP Media I and AIM V (Invitrogen). Media was supplemented with 6000 I.U./mL rhIL-2 on days 2 and 5 of the 8 day REP. This protocol was also implemented in T75 flasks at exactly triple the aforementioned cell counts and reagents.

### Expansion of CD8^+^ TIL

CD8^+^ T cells were enriched from propagated TIL by depletion of CD4^+^ cells with a human CD8^+^ isolation kit (Miltenyi Biotech, Germany) according to the manufacturer’s protocol. CD8^+^ TIL were expanded for 8 days using a mini-REP in T25 flasks.

### Flow cytometry

Excess tumor tissues not used for TIL generation were digested physically using scalpels and enzymatically with media containing collagenase type IV (1 mg/mL), DNase type IV (30 U/mL), and hyaluronidase type V (100 μg/mL) (Sigma). Single cell suspensions were strained and counted via trypan blue exclusion, followed by cryopreservation for future analysis. Tumor digests and TIL were stained at varying points in the expansion process using the following: APC, PE, FITC, PECy7, PerCpCy5.5, and AlexaFluor700 conjugated antibodies: CD3, CD4, CD8, CD25, FoxP3, CD69, PD-1, GITR, 4-1BB, CTLA-4, CCR7, CD45RO, CD27, CD28, and CD122 (BD Biosciences, San Jose, CA; , eBioscience, La Jolla, CA; or R&D Systems, Minneapolis, MN). All panels contained the LIVE/DEAD Aqua or LIVE/DEAD Near-IR dead cell stain (Invitrogen). Intracellular Foxp3 staining was carried out in accordance with the manufacturer’s protocol (eBioscience). Data were acquired on an LSRII flow cytometer (BD Biosciences) and analyzed using FlowJo software (TreeStar, Inc.).

### CD107a and IFN-γ functional assay

A flow cytometry-based analysis of TIL function was designed to detect CD107a membrane expression and intracellular IFN-γ expression. Briefly, TIL were stimulated at 37 °C overnight at a concentration of 5 × 10^5^ cells/mL in the presence of 25 ng/mL phorbol myristate acetate (PMA) and 0.5 μM ionomycin. After 90 min of stimulation, APC conjugated CD107a or its IgG1 k isotype (BD Biosciences) were added. Brefeldin A, at 1 μg/mL, (Sigma Aldrich) and monensin, at 0.067 % (v/v), (GolgiStop, BD Biosciences) were added one hour later. After 18 h, cells were washed once with Staining Buffer (PBS containing 0.05 % (v/v) Human AB serum), then surface stained (4 °C for 30 min.) with AlexaFluor 700, PECy7, and PerCpCy5.5 conjugated anti-CD3, CD4, and CD8 (BD Biosciences), respectively. The LIVE/DEAD Aqua (Invitrogen) dye was included as a cell viability stain. Cells were washed once with Staining Buffer and then fixed and permeabilized with BD Cytofix/Cytoperm for 20 min at 4 °C and washed twice with BD Perm/Wash (BD Biosciences). Intracellular staining of FITC conjugated anti-IFN-γ (BD Biosciences) occurred at 4 °C for 30 min, followed by one wash with BD Perm/Wash. Cells were stored in 0.5 % paraformaldehyde (Thermo Fisher Scientific) at 4 °C in the dark. All samples were run on an LSRII flow cytometer (BD Biosciences) within 48 h and analyzed using FlowJo software (TreeStar, Inc.)

### Co-culture assays

Effector TIL were co-cultured in TIL CM in round-bottom 96-well plates (5 × 10^4^ TIL) at a 1:1 ratio with pancreatic cancer cell line targets for 24 or 48 h. Cell lines MiaPaca-2, Panc-1, CFPAC-1, and HPAF-II were obtained from the ATCC and cultured according to the manufacturer’s guidelines. Supernatants were collected and reactivity of pancreatic TIL to HLA-matched pancreatic cancer cell lines was determined following the manufacturer’s instructions for the IFN-γ ELISA assay (Human IFN-γ Quantikine ELISA Kit, R&D Systems). Optical density of each well was measured at 450 nm and IFN-γ concentration was calculated from the standard curve.

### Viability analysis

TIL were counted and assessed for viability using the Guava ViaCount Reagent and ViaCount software according to the manufacturer’s instructions and analyzed on the Guava PCA system (Guava Technologies, Hayward, CA). Alternatively, TIL were counted by trypan blue exclusion on a hemocytometer.

### Statistical analysis

Data represented as scatter plots show individual patient data points or fragments where indicated as well as error bars representing mean values and the standard deviation (SD) or standard error of the mean (SEM). *P* values were determined using GraphPad Prism software by the paired, two-tailed *t*-test, Wilcoxon signed rank test, Mann Whitney test, or unpaired Student’s *t*-test where indicated.

## Additional file

Additional file 1: Figure S1. Pancreatic TIL cultured in the presence of PD-1 blocking antibody demonstrated increased tumor reactivity. TIL expanded with anti-PD-1 or the isotype control were co-cultured with HLA-A matched (black) and mismatched (empty) tumor lines. IFN-gamma release was assessed by ELISA after 24 h and reported as mean ± SD (*n* = 1). **Figure S2.** Pre-REP and Post-REP Pancreatic TIL are phenotypically similar. Pancreatic TIL were expanded from fragments in IL-2, then subjected to the full, two week REP. TIL were stained for the indicated surface markers, in addition to CD3, CD4, CD8, and a viability dye. Data represent percentage positive of the parent gate CD4 (A) or CD8 (B) as a mean ± SD (*n* = 3). (PPTX 285 kb)

## Abbreviations

ACT: Adoptive cell therapy
GI: Gastrointestinal
PMA: Phorbol myristate acetate
SD: Standard deviation
SEM: Standard error of the mean
TIL: Tumor infiltrating lymphocytes
REP: Rapid expansion protocol
