# Supplementary material for: Expansion of tumor-infiltrating lymphocytes (TIL) from human pancreatic tumors
Source: J Immunother Cancer. 2016 Oct 18;4:61. doi: 10.1186/s40425-016-0164-7 (PMC5067894; doi:10.1186/s40425-016-0164-7)
Supplement: Additional file 1: Figure S1. — Pancreatic TIL cultured in the presence of PD-1 blocking antibody demonstrated increased tumor reactivity. TIL expanded with anti-PD-1 or the isotype control were co-cultured with HLA-A matched (black) and mismatched (empty) tumor lines. IFN-gamma release was assessed by ELISA after 24 h and reported as mean ± SD (n = 1). Figure S2. Pre-REP and Post-REP Pancreatic TIL are phenotypically similar. Pancreatic TIL were expanded from fragments in IL-2, then subjected to the full, two week REP. TIL were stained for the indicated surface markers, in addition to CD3, CD4, CD8, and a viability dye. Data represent percentage positive of the parent gate CD4 (A) or CD8 (B) as a mean ± SD (n = 3). (PPTX 285 kb) [file 40425_2016_164_MOESM1_ESM.pptx]

## Slide 1
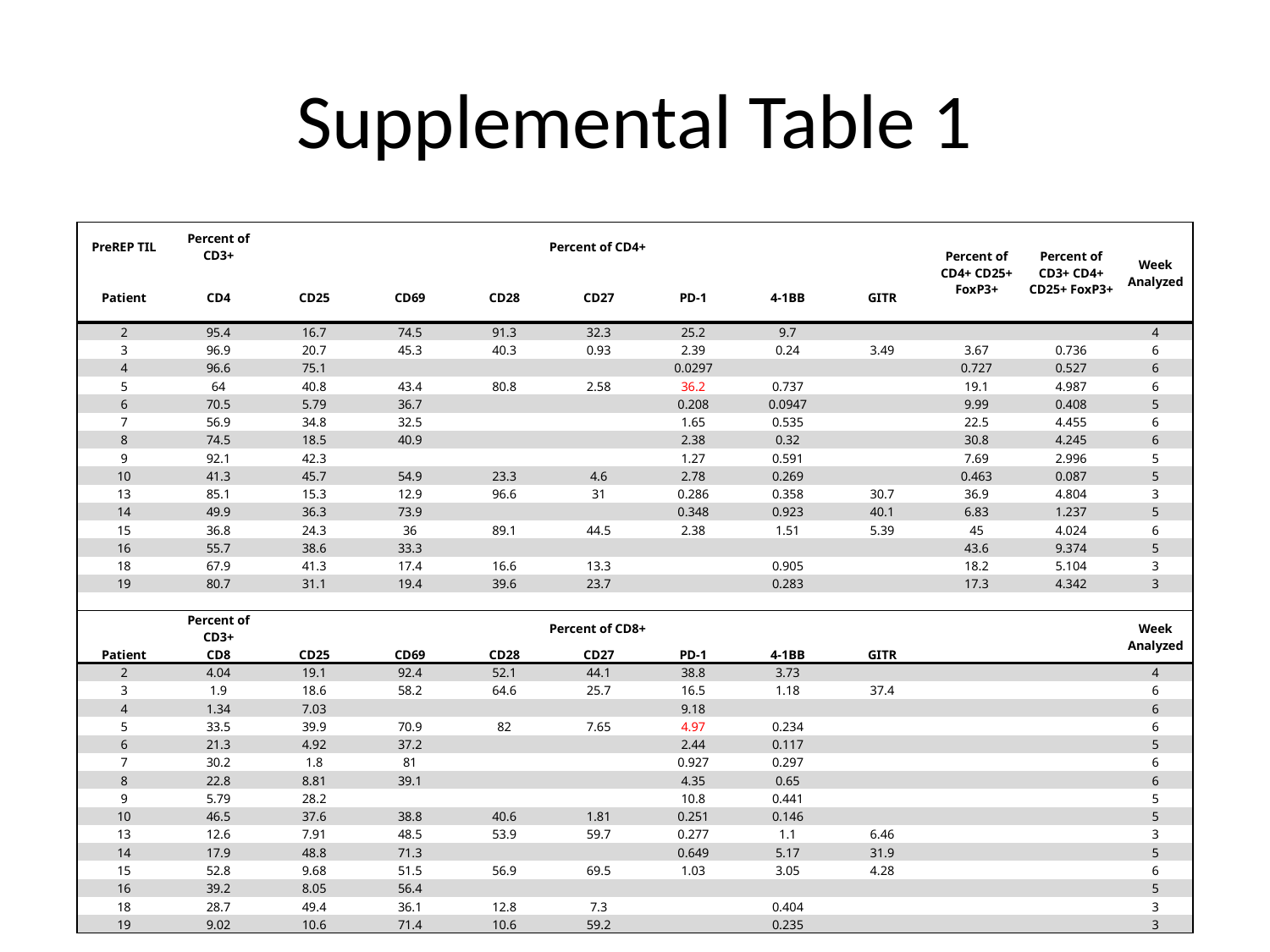

# Supplemental Table 1
| PreREP TIL | Percent of CD3+ | Percent of CD4+ | | | | | | | Percent of CD4+ CD25+ FoxP3+ | Percent of CD3+ CD4+ CD25+ FoxP3+ | Week Analyzed |
| --- | --- | --- | --- | --- | --- | --- | --- | --- | --- | --- | --- |
| Patient | CD4 | CD25 | CD69 | CD28 | CD27 | PD-1 | 4-1BB | GITR | | | |
| 2 | 95.4 | 16.7 | 74.5 | 91.3 | 32.3 | 25.2 | 9.7 | | | | 4 |
| 3 | 96.9 | 20.7 | 45.3 | 40.3 | 0.93 | 2.39 | 0.24 | 3.49 | 3.67 | 0.736 | 6 |
| 4 | 96.6 | 75.1 | | | | 0.0297 | | | 0.727 | 0.527 | 6 |
| 5 | 64 | 40.8 | 43.4 | 80.8 | 2.58 | 36.2 | 0.737 | | 19.1 | 4.987 | 6 |
| 6 | 70.5 | 5.79 | 36.7 | | | 0.208 | 0.0947 | | 9.99 | 0.408 | 5 |
| 7 | 56.9 | 34.8 | 32.5 | | | 1.65 | 0.535 | | 22.5 | 4.455 | 6 |
| 8 | 74.5 | 18.5 | 40.9 | | | 2.38 | 0.32 | | 30.8 | 4.245 | 6 |
| 9 | 92.1 | 42.3 | | | | 1.27 | 0.591 | | 7.69 | 2.996 | 5 |
| 10 | 41.3 | 45.7 | 54.9 | 23.3 | 4.6 | 2.78 | 0.269 | | 0.463 | 0.087 | 5 |
| 13 | 85.1 | 15.3 | 12.9 | 96.6 | 31 | 0.286 | 0.358 | 30.7 | 36.9 | 4.804 | 3 |
| 14 | 49.9 | 36.3 | 73.9 | | | 0.348 | 0.923 | 40.1 | 6.83 | 1.237 | 5 |
| 15 | 36.8 | 24.3 | 36 | 89.1 | 44.5 | 2.38 | 1.51 | 5.39 | 45 | 4.024 | 6 |
| 16 | 55.7 | 38.6 | 33.3 | | | | | | 43.6 | 9.374 | 5 |
| 18 | 67.9 | 41.3 | 17.4 | 16.6 | 13.3 | | 0.905 | | 18.2 | 5.104 | 3 |
| 19 | 80.7 | 31.1 | 19.4 | 39.6 | 23.7 | | 0.283 | | 17.3 | 4.342 | 3 |
| | | | | | | | | | | | |
| | Percent of CD3+ | Percent of CD8+ | | | | | | | | | Week Analyzed |
| Patient | CD8 | CD25 | CD69 | CD28 | CD27 | PD-1 | 4-1BB | GITR | | | |
| 2 | 4.04 | 19.1 | 92.4 | 52.1 | 44.1 | 38.8 | 3.73 | | | | 4 |
| 3 | 1.9 | 18.6 | 58.2 | 64.6 | 25.7 | 16.5 | 1.18 | 37.4 | | | 6 |
| 4 | 1.34 | 7.03 | | | | 9.18 | | | | | 6 |
| 5 | 33.5 | 39.9 | 70.9 | 82 | 7.65 | 4.97 | 0.234 | | | | 6 |
| 6 | 21.3 | 4.92 | 37.2 | | | 2.44 | 0.117 | | | | 5 |
| 7 | 30.2 | 1.8 | 81 | | | 0.927 | 0.297 | | | | 6 |
| 8 | 22.8 | 8.81 | 39.1 | | | 4.35 | 0.65 | | | | 6 |
| 9 | 5.79 | 28.2 | | | | 10.8 | 0.441 | | | | 5 |
| 10 | 46.5 | 37.6 | 38.8 | 40.6 | 1.81 | 0.251 | 0.146 | | | | 5 |
| 13 | 12.6 | 7.91 | 48.5 | 53.9 | 59.7 | 0.277 | 1.1 | 6.46 | | | 3 |
| 14 | 17.9 | 48.8 | 71.3 | | | 0.649 | 5.17 | 31.9 | | | 5 |
| 15 | 52.8 | 9.68 | 51.5 | 56.9 | 69.5 | 1.03 | 3.05 | 4.28 | | | 6 |
| 16 | 39.2 | 8.05 | 56.4 | | | | | | | | 5 |
| 18 | 28.7 | 49.4 | 36.1 | 12.8 | 7.3 | | 0.404 | | | | 3 |
| 19 | 9.02 | 10.6 | 71.4 | 10.6 | 59.2 | | 0.235 | | | | 3 |

## Slide 2
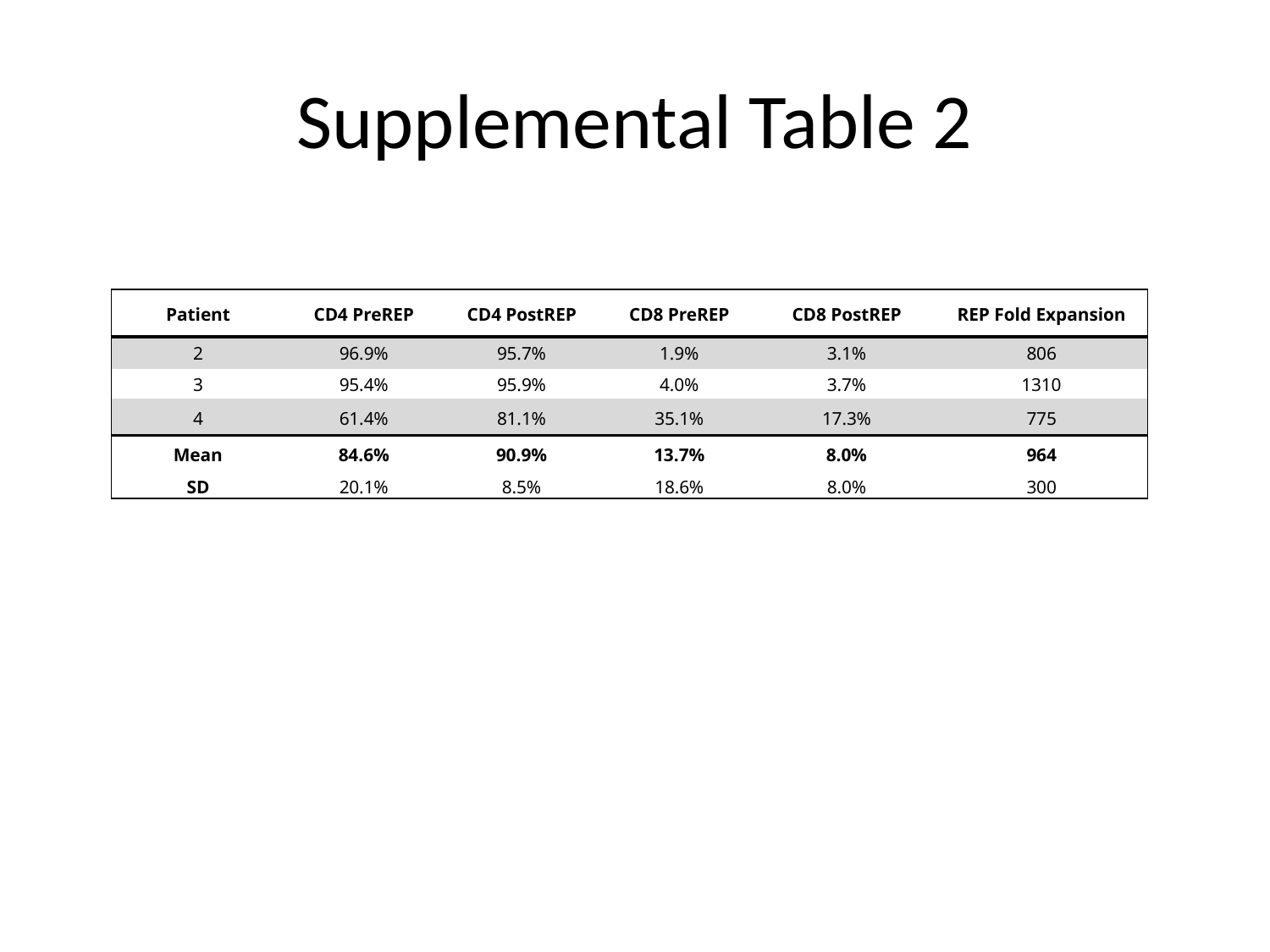

# Supplemental Table 2
| Patient | CD4 PreREP | CD4 PostREP | CD8 PreREP | CD8 PostREP | REP Fold Expansion |
| --- | --- | --- | --- | --- | --- |
| 2 | 96.9% | 95.7% | 1.9% | 3.1% | 806 |
| 3 | 95.4% | 95.9% | 4.0% | 3.7% | 1310 |
| 4 | 61.4% | 81.1% | 35.1% | 17.3% | 775 |
| Mean | 84.6% | 90.9% | 13.7% | 8.0% | 964 |
| SD | 20.1% | 8.5% | 18.6% | 8.0% | 300 |

## Slide 3
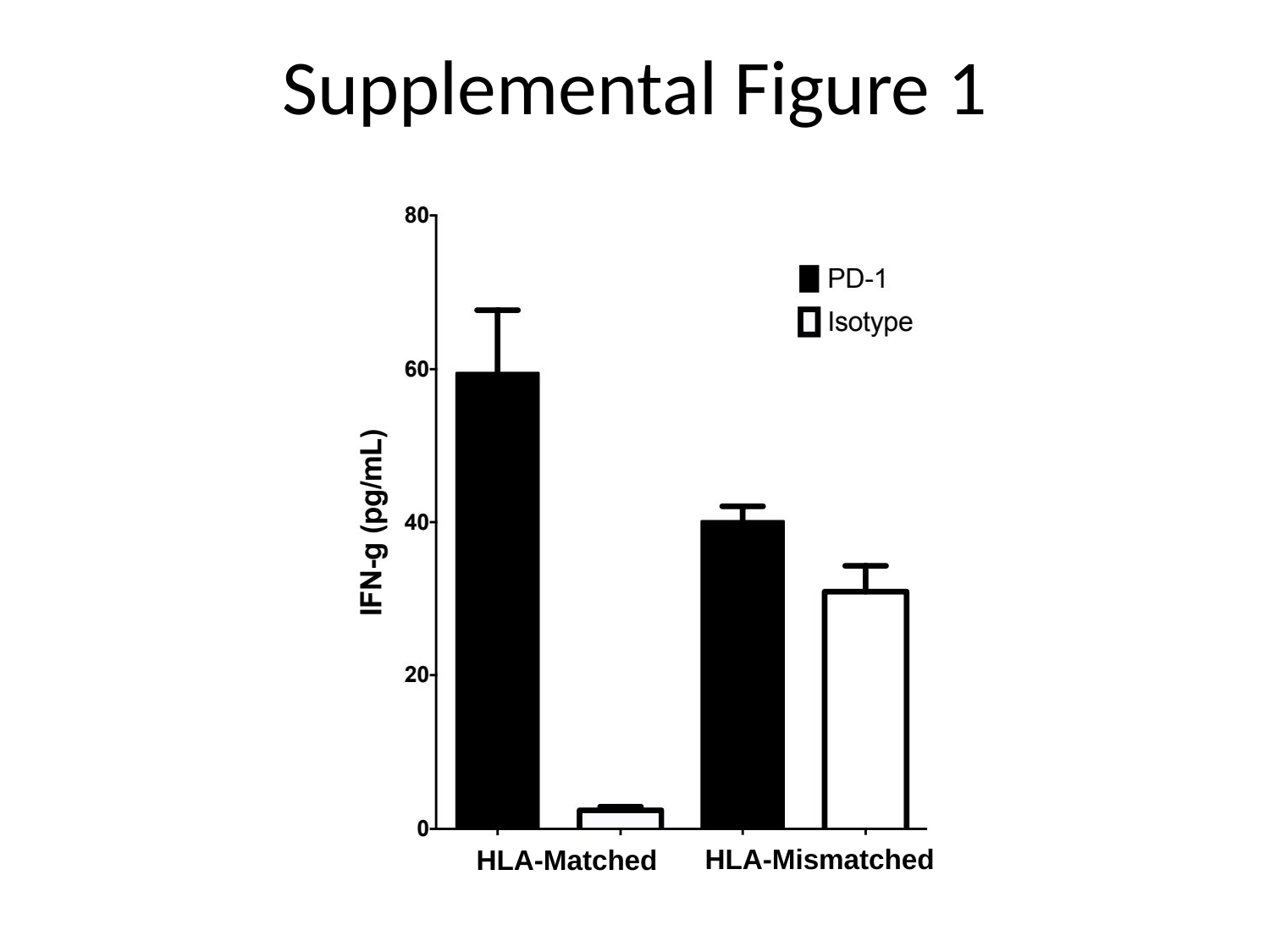

# Supplemental Figure 1
HLA-Mismatched
HLA-Matched

## Slide 4
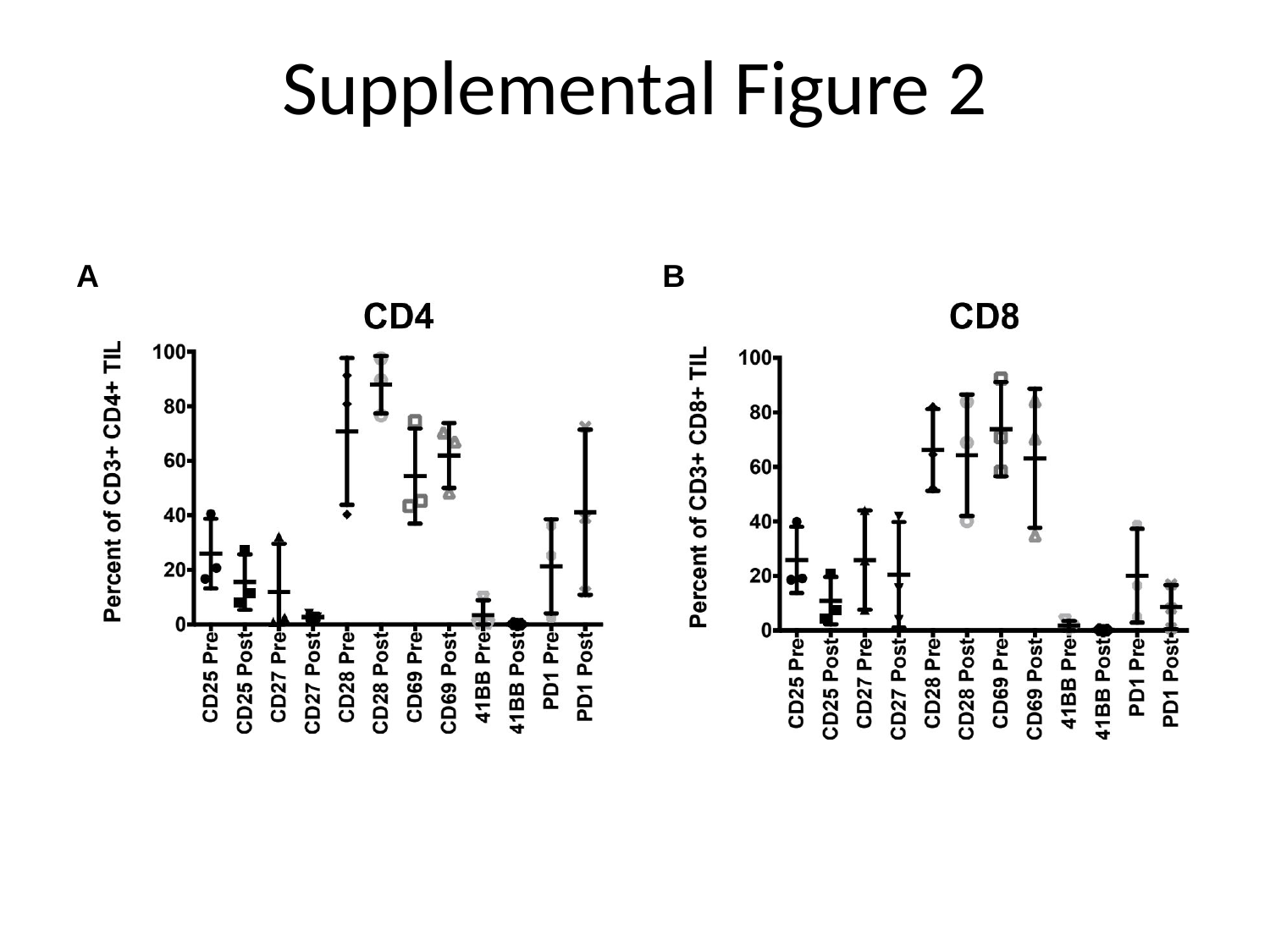

# Supplemental Figure 2
B
A
